# Notices

**Published:** 1866-10

**Authors:** 


### NOTICES.

We do not remember to have read in many a day any article on any subject with such an intense interest as Tischendorf's description of the manner in which he became possessed of the Sinai Bible, in one of the cloisters of Mount Sinai, it being, perhaps, the oldest copy in the world of the old and new Testaments written in Greek about the year 333, A. D.

"I had rather," said a venerable and courtly man, "have discovered for the Queen of England the Sinaitic manuscript, than "Koh-i-nur;" and certainly it is worth more to the human race than any number of Koh-i-nurs, because it is a "mountain of light" not yielded by a perishable jewel of an hour, but a source of light upon the Holy Scripture, which shall not cease to shine during the ages. It was translated from the author's German narration for the *Hours at Home*, a New York publication devoted to religion and useful literature. Thirty cents will procure a copy containing the article, sent to Charles Scribner & Co., 654 Broadway, New York, it being the June No. for 1866, being No. 2 of volume third.

Who in the wide world but a German scholar could start from home with a hundred Dutch dollars and his only coat unpaid for, on an uncertain mission which was to take him through three continents at a cost of three thousand dollars and fifteen years' time. But the plodding Dutchman did it; and what is more, gloriously succeeded, to the great joy of Pope, Autocrats and Kings.

The Christian puplic owe much to the enterprise of *Hours at Home* in obtaining this translation of Tischendorf's absorbing narrative.

The Dental Register, edited and puplished monthly at Cincinnati, Ohio,—$ 3 a year—by J. Taft, is well worth the patronage of the Dental profession. It is now near the close of its 20th volume.

The Chicago Medical Examiner, a Monthly, $ 3 a year; edited by Professor Davis, has a most important article in the June number on the Primary Surgery of Gen. Sherman's Campaigns, by Professors Andrews and Wentworth. Dr. R. P. Cotton of London, on spitting blood as a sign of Consumption. Our own views are that if a man ever spits blood, he will in nine cases out of ten die of consumption unless promptly and properly treated. Although friends and physicians are too ready to ascribe it to any other cause, it is infinitely safer to say, that when a man spits blood it means death within two years, with very rare exceptions; especially when there is cough and the cough always gets better after each bleeding. On the other hand when a woman spits blood we consider it a sign of no special importance of itself, unless it be a sign of their going to get well. ——Also an article on the newly discovered cause of Fever and Ague, by Prof. J. H. Salsbury, who has immortalized himself by the discovery, if time verifies it. The idea is that the cause of fever and ague, for example, is a vegetable seed, of which the air is full at the surface of the earth at sunrise and sunset. None are found in the air during the middle of the day, nor at any time usually at a greater height than thirty feet; answering precisely to the laws of miasm, published by us several years ago.—(See articles "Miasm," and "Month Malign" and "Farmers' Houses," in previous volumes of the JOURNAL.) It is one of the most important of all practical subjects connected with disease and the use we have made of the facts known in regard to maladies of this character in the shape of Spring and Autumnal diseases, epidemics, etc., was to recommend as an absolute and infallible preventive of these ailments in any family in the Autumn was:

First — To sleep in the upper stories.

Second — To exclude the night air from the chambers.

Fourth.— Never go outside the house till breakfast was eaten.

Fourth.— Come into the house before sundown and not go out afterwards, at least until supper was taken.

Fifth.— Kindle a fire in the family sitting-room at sunrise and sunset, and sit by it. All these items correspond with Professor Salisbury's discovery. That these sporules are not found in the air of hot noon-day is because heat is incompatible with their presence.

BY the thoughtful courtesy of Senator Morgan of New York City, we are in receipt of a volume of "Statistics of the United States," including Mortality, Property, Army and Navy, Deaf and Dumb, Banks, Railroads, Public Press, Churches, Educational, etc., in 1860; being the final exhibit of the eighth census, under the direction of Hon. James Harlan of Iowa, Secretary of the Interior. It is a quarto volume of 584 pages, replete with the most important information connected with our government, and will be a standard book of reference of inestimable value.

TO PURIFY RANCID LARD. — The following is said to effect this perfectly:— "Knowing the antiseptic qualities of the chloride of soda, I procured three ounces, which I poured into a pailful of soft water, and when hot,the lard added. After boiling thoroughly for an hour or two it was set aside to cool. The lard was taken off when nearly cold, and subsequently boiled up. The color was restored to an alabaster white, and the lard was as sweet as a rose." Rancid butter may be treated in the same way.

THE PULSE.—In ascending into the air, the pulse, for the first mile or two, beats, for each hundred yards of ascent, one faster thus, if it beats seventy times in a minute, which is the average for adults in health, it will beat at the height of one mile, about eighty-eight times in a minute. We have not heard it so stated, but the breathing must increase with proportional rapidity, for the pulsations at the wrist, or the beating of the heart, which are at the same instant, are in the proportion to the breathing of four to one in health. It does not appear to require much effort to draw a breath, but Mr. Fitch has estimated that the power expended in breathing during twenty-four hours would raise a hundred pounds to the height of seven hundred feet.

A DOSE.—The following is taken from a newspaper. The stupidity which could recommend such an abominable conglomeration is amazing. What is worthy of note, we see the same recommendation in the same paper year after year, as regularly as the tomatoe season comes round:

"For a family of half a dozen persons, take six eggs, boil four of them *hard*, dissolve the yolks with vinegar sufficient, add about three teaspoons of mustard and mash as soon as possible; then add the two remaining eggs, (raw) yolk and white, stir well; then add salad oil to make altogether sauce sufficient to cover the tomatoes well: add plenty of sauce and cayenne pepper, and beat thoroughly until it frosts. Skin and cut the tomatoes a full fourth of an inch thick, and pour the sauce over."

The dish may be a very palatable one to any drunkard or tobacco-chewer, whose faculties of taste have been so blunted that nothing but fire and brimstone could wake them up to any sensibility. A curious item in reference to the dose aforesaid is, that it gives the exact proportion of every item except the quantity of tomatoes to be used; is it a pint or a bushel? What is more surprising is, that the article is headed "Tomatoes for supper." They will live longest whose food is simple, and the fewer different articles we use at any meal beyond three or four, the better; while a simple supper of a piece of plain cold bread and butter with a glass of water or a cup of warm drink of any kind, will be of incalculable benefit in the course of a lifetime to all persons who spend most of their time indoors. It is variety which tempts us all to exceed at meal-times, and they are wisest and will be the healthiest and happiest, and the longest lived by a score of years at least, who begin early to have simple tastes, and feed at each meal on one meat, one vegetable, and one furit, with any suitable drink, with bread, butter, salt, vinegar, pepper, and such salads as desired. There may not be found one family in a thousand willing to adopt such a course, but it would prevent a large amount of pain and suffering, and wasting sickness, if something of an approximation was made to such a dietary, and as the quantity is not limited, there is no danger of starvation, especially as the articles may be changed at each meal, which allows of abundant variety.

ICE PRESERVER.—Make a double bag with a space of three inches between the inner and outer one, fill this space with feathers, at side and bottom, as tight as can be packed, and if the mouth is closely tied, a few pounds of ice will keep a week.

BOSTON is having a sensation—or dispensation—growing out of the adveut of Professor Blot, whose lectures on the art of cooking attracted so much attention in New York. The Post is in ecstasies after the following fashion; "Professor Blot's classes in Mercantile Hall, are filling up rapidly. More of the positive science and system of cookery is now talked about among matrons, young and old, than was ever imported into a year's conversation before. Many a hard working man of business experiences a sense of relief at the change in his domestic menage. His head is clearer; his heart is lighter; his home wears a pleasanter look. Professor Blot has done it. Thanks to the welcome lecturer on the mysteries of the culinary art, who brings happiness as well as better dishes and a sounder digestion."

THE AMERICAN TRACT SOCIETY, 150 Nassau Street, New York, have issued eighty questions on cards, with Bible answers, thus: "Should we be kind to each other?" answer, Eph, 4.32. "Be ye kind to one another." In this way most important practical information is communicated to children in an easy manner, with Scripture authority therefore. Also a paper-covered 24mo. 48 pages, "To those commencing a religious life." This is a useful work to that too much neglected class of persons, those who have just joined the church; the untiring zeal which some show in getting persons to join the church and then dropping them like a hot potatoe, as if salvation were secured, is a too common and a very grave fault. The difficulties and discouragements of those just entering a religious life are not a whit less than those which beset them previous to making a profession of religion; it is like throwing a baby in a mudhole and let it waddle out the best way it can. To these unfortunate forsaken ones, this three cent book is sweetly encouragiug, and very instructive. We don't mean to treat so serious a subject irreverently, but hope that the manner will attract the greater attention of those whose duty it is to carry the lambs in their bosom.

THE REV. CHARLES PEABODY'S narration of "Twenty years among the Colporteurs," will encourage working christians, while it will shame those who expect to reach heaven by lolling and lounging

"On flowery beds of ease,"

and to whom the whole of religious duty seems to be in riding to church on Sunday morning in splendid equipages, with a "love feast" following, in the shape of a Sunday dinner with choice friends, from which they arise, in the identical language we heard from the mouth of that venerated man, the Rev. James W. Alexander, "more like gorged anacondas than anything else," seeming to forget that the compliment of practical piety is not made up by large donations of money, which do not involve one single, the slightest self-denial, but that more precious gifts to the treasures of the Lord is found in individual personal efforts, requiring mental anxiety, bodily fatigue, and trying self-denial.

Come to think of it, perhaps we had better change our "line" in spite of General Grant's example, and go to writing sermons or "Health Tracts for the Soul. Wonder if it would be a more profitable investment of brain-work, bring in more money, to buy candy for our children now, alas! all in their teens; our once little Alice, just having passed twelve, yet towering up to the heighth of five feet three, and still "going ahead," bless her little heart!

The same society have also printed "Among the Willows," or "How to do Good," by J. H. Tangille, being the history of Mary Cludge. The leading incident of this narrative having occurred at an institution of learning a few years since..

"The Awakening of Italy," by the Rev. J. A Wylie, D.D., 12 mo. 364 pages, from personal observations during four visits to Italy, including a year's residence in that country. Every zealous friend of evangelical religion ought to get the book and read it; it is of absorbing interest.

"Sisters and not Sisters," by Mrs. M. E. Berry, 246 pps., 12 mo. Being lessons drawn from actual scenes in single home life, best suited to our common wants, a book which might be profitably read half a dozen times to the family circle before the winter fireside.

"The young Lady of Pleasure," anonymous, without preface, 316 pp. 12mo. A series of thirty letters to young ladies, closing with "Your affectionate friend, M. Stanley." These are letters written by a lady teacher, at the request of a former pupil, who had "finished her education" in the usual meaning of the phrase, but who finds, to her surprise, that she is just entering upon the practical duties of life, and has just entered "The Practical Knowledge class," the previous classes having been theoretical, preparatory, having done little more than learning the mind one lesson, how to think. The motto on the title-page from Young, is alone, suggestive of a volume.

"False pleasures from Moed her joys impart,
Rich from within, and self-sustained the true."

This is one of the fittest works we have lately seen for a girl just leaving school.

A Doctor's Life.—There is more than money's worth in a letter like the following from a Lady, the offspring of a gratitude living in its freshness twenty years after its birth. "I am the wife of A. P. R. whom you treated for lung disease about twenty years ago, and were the means of arresting and curing a malady which we thought would carry him to an early grave. My husband is now forty-three years old, and is very healthy. (Now for the political.) He has gone through five years service in the war, on the Union side, of course, for no such clever man as Capt. A. P. R. could ever be a "Reb."

Mountain Weight.—A mountain is pretty heavy physically, and the expression is used to indicate the oppressed condition of the mind. Physicians are repeatedly consulted by persons who think they are "in a very bad way" when there is actually no physical derangement, but the trouble has arisen from a misapplication, or misapprehension of the meaning of facts and occurrences. A young gentleman writes, "I feel no anxiety now, you have lifted a most wearisome burden from my mind. I cannot tell you how strong and well I feel; time passes cheerfully and swiftly, while before, only a day of life was a dread to me, not from actual trouble, but from fear of the future.

Hair-Wash.—The very best and most efficient hair-wash in the world is soap-suds rubbed thoroughly into the scalp with the ends of the fingers; then rinse well with pure water, and wipe dry with a towel. If this is done thoroughly, once a week, there will be found scarcely a particle of dandruff, and the hair will be soft and silken, with the appearance of having more of it; any hair-oil or pomatum whatever, is a filthiness and a hair destroyer, as they gather dust, obstruct the pores of the scalp, prevent the access of the air to the roots of the hair, and rots them.

Rats.—The odor of dead rats induces disease in a whole household, while most rat poisons are fatal to the family. If the bisulphide of carbon is poured into their holes it will drive them from the premises in twenty-four hours; the next best remedy is a rat-trap baited with toasted cheese.

Purifying Water.—A plum-sized lump of alum attached to a string and swung around a few times slowly through a pitcher of water will cause the sediment to fall to the bottom in a few minutes. The neutral sulphate of alumine will make lime-water perfectly pure, destroying at the same time all organic compounds; almost all water has lime in it.

COOKERY.—The severest blow to intelligence offices is Pierre Blot, (pronounced Blow) the lecturer on cookery. The Professor is both a scholar and a gentleman; and every housewife who has an opportunity, by attending a full course of lectures, will, in spite of herself, become deeply interested in it, and will acquire an amount of practical information which will be useful for life, and which, if utilized, will save the health and money of the household.

TOBACCO.—Four hundred millions of pounds of tobacco was raised in the United States during the year 1860. More than half of which was produced in the Northern States; it ruins the health of many, and is the first step towards drunkenness, in millions of cases, and not only makes water insipid, but creates a desire for something to drink, which only spirits can satisfy.

PHYSIOGNOMY teaches how to know a man by his face. Just published, "New Physiognomy, or Signs of Character," 768 pages, 8vo. sent by mail for $5.00, post-paid, by Fowler and Wells, 389 Broadway, New York. It has more than a thousand illustrations of the faces of remarkable men, and is one of the most interesting books which has lately come to our notice; it is eminently practical; it enables one to judge of human nature through the temperament, external forms, and the face; to do this well, is an important element of success in life, in every department, from the school teacher to the general; from the parent to the czar; whoever has to employ a servant, or a helper, one or a thousand, will fail or succeed according to the correctness with which he has judged of their character. It is a book suited to every parlor-table in the land, as it can be taken up with profit even if but for a moment. Faces change with the character, and employment, see Abraham Lincoln; love signs, transmitted features; hands and feet, gait, &c.

THE AMERICAN NEWS CO., 121 Nassau St., New York, W. B. Zeibur, Philadelphia, Henry Taylor, Baltimore, and Morrison, Washington City, have for sale the Post-Office Directory for 1866. It contains the names of all the post-offices and post-masters names in the United States and Canada, with names of new post-offices alphabetically arranged; with rates of foreign and domestic postage; lists of money-order post-offices with table of distances by the shortest mail routes from the county seats, from Washington, revised and corrected by J. Disturnell. It is sent post-paid, for $1.50; its value and usefulness to business and professional men is seen at a glance.

CRITICISM.—"We often think that Dr. Hall is a trifle too sticklish about small matters, and apt to magnify a molehill of hygenic neglectfulness into a mountain of trouble-making,—and in some matters too nearly an extremist; but if he errs, it is always on the safe side."

We do not like the above criticism, at all, because it is of no possible use to us or our readers; if the editor of the Lyons Republican will state in what we are too minute or are over particular in small matters, we will investigate and correct the fault, for that is the only advantage of criticism; if he will only specify, we promise to have some fun at his expense, and the profit of our subscribers.

New-England Dying Out.—About three-fourths of all the children born in Boston during 1865 were of parents born in a foreign land; therefore, argues one of the papers, "the Yankee stock will in time die out in New-England." We think by that time, Yankee stock will have peopled, will be the predominant stock of this continent, from the St. Lawrence to the Gulf, and from the Atlantic to the Pacific shore. Because Yankee intelligence knows how to rear children to maturity, while foreign ignorance and filth kills, almost as soon as born, for out of every hundred children dying, eighty-eight are of foreign parentage, and this has been the rate for the last five years in New York city, where the native population is 49 per cent, and the foreign 51, or nearly equal, and it is presumed that the same proportions hold good in all our large cities. Hence if 62 per cent of all the children born in Boston are of foreign parents, and 88 per cent die, it is very easy to see they might as well not have been born at all, and a great deal "mightier," as far as the question of the foreign outstripping the Yankee stock is concerned. The fact is, neither Yankee men, nor Yankee principles, nor Yankee thrift, will ever die out, while this planet is inhabited; and if it is ever depopulated by a conflagration, the last survivor of a smouldering world will be Jonathan, at the death, singly and alone, resolutely trying to put out the fire; if by famine, the last loaf of bread will be owned by a Yankee.

Advertisements.—Our readers will take notice, once for all that the mere admission of an advertisement does not involve any opinion whatever in its favor. Every one must try for himself, and hold on to that which is good, but expect to be bitten sometimes.

